# Female Mate Choice in Wild Kenyan Blue Monkeys (*Cercopithecus mitis*)

**DOI:** 10.3390/ani14111589

**Published:** 2024-05-28

**Authors:** Sofia Schembari, Caitlin Miller, Su-Jen Roberts, Marina Cords

**Affiliations:** 1 Department of Ecology, Evolution & Environmental Biology, Columbia University, 10th Floor, Schermerhorn Extension, 1200 Amsterdam Avenue, New York, NY 10027, USA; sms2372@columbia.edu (S.S.); caitlin3039@gmail.com (C.M.); sujen.roberts@gmail.com (S.-J.R.); 2 The New York Consortium in Evolutionary Primatology, New York, NY 10065, USA

**Keywords:** mate choice, female choice, paternity, intersexual power, blue monkeys, *Cercopithecus mitis stuhlmanni*

## Abstract

**Simple Summary:**

Historically, researchers’ biases about male and female behavior have affected the study of mate choice (animals’ selective responses to certain sexual partners over others), and female mate choice has often been dismissed as unimportant. In this study, we investigated whether female blue monkeys could exercise mate choice successfully by siring offspring with their preferred males. Blue monkeys generally live in a one-male multi-female social organization, but multiple males may join the group during the breeding season. Female blue monkeys, thus, have access to multiple potential mates, and initiate sexual interactions more than males, making them an appropriate species in which to study female choice. We found that a female’s most preferred male was most likely to be the father of her infant, even if he was not a consistent group member. Though competition between males may still influence paternity outcomes, these results suggest that blue monkey females can exercise mate choice successfully, even in a social organization that would seem to favor male control.

**Abstract:**

Female mate choice may drive sexual selection, but discerning whether female behaviors reflect free expression of choice or responses to constraints can be difficult. We investigated the efficacy of female choice in wild blue monkeys using 10 years of behavior and paternity data (N = 178 male–female dyads). Although blue monkeys live modally in one-male polygynous groups, where male-biased intersexual power is expected, females can access multiple potential mates during seasonal male influxes and occasional intergroup encounters. Additionally, extra-group males sire offspring. We examined female resistance rates to male-initiated sexual interactions, and unsolicited proceptive behavior that females directed to males (corrected for male availability). Females seldom resisted male solicitation, but initiated sexual interactions more than males. Females generally preferred residents. Those who preferred non-residents tended to have residents with longer tenures, but neither female parity nor rank influenced the tendency to prefer non-residents vs. residents. The male most solicited by a particular female fathered that female’s infant 82% of the time; odds of siring were 26 times higher for most vs. nonpreferred males. Female preference predicted paternity even more strongly among non-resident males, with odds of siring 33 times higher for most vs. nonpreferred non-residents. Neither female rank nor parity influenced her likelihood of having her preferred partner as sire. Paternity by preferred males did not affect infant survival. While we cannot fully discount the effect of male–male competition on paternity, these results suggest that blue monkey females can exercise choice successfully, even in a polygynous mating system.

## 1. Introduction

Mate choice, animals’ selective responses to certain potential sexual partners over others, is a common phenomenon across the animal kingdom, but the study of mate choice has historically been burdened with researchers’ biases about how males and females should behave, and which sex should have control over reproductive outcomes. In fact, mate choice can take many forms, and can be exhibited by both females and males [1]. Female mate choice has often been disregarded as a significant driver of sexual selection in mammals, where males have been assumed to hold greater reproductive control [2]. Primates, in particular, tend to have male-biased power, related to sexual dimorphism in body mass and unequal sex ratios [3]. Accordingly, female reproductive control, whether through mate choice, resistance, or promiscuity, is generally expected to be limited [4]. Although previous studies have attempted to show that female sexual behaviors, such as proceptivity and resistance, can effectively influence copulations and paternity in mammals (e.g., Alaskan moose (*Alces alces gigas*) [5]; capybaras (*Hydrochoerus hydrochaeris*) [6]; chimpanzees (*Pan troglodytes verus*) [7]; gray-cheeked mangabeys (*Lophocebus albigena johnstoni*) [8]; Tonkean macaques (*Macaca tonkeana*) [9]; tuco-tucos (*Ctenomys talarum*) [10]; but see [11]), it can be difficult to assess whether observed female behavior results from free expression of choice, or from female responses to constraining factors [12]. 

For female mate choice to occur, a female must first have access to multiple possible partners that differ in perceived quality, motivating her to select the best potential sire from several options [13]. Social organization could limit a female’s access to mates: for example, a solitary or pair-living female who does not interact with more than one male during a single fertile period would not be able to exercise mate choice. Females living in uni-male groups are expected to have limited mate choice opportunity for similar reasons, but additionally because of the superior physical power of males in the contest-based mating system that typically coincides with this social arrangement [4]. By contrast, females that live in multi-male groups should have more opportunities to interact and mate with multiple males. It is important to remember, however, that for group-living females, potential partners could also come from outside the group. Extra-pair or extra-group paternity has been detected in many species, even in those that live as pairs or in one-male groups [14,15,16]. Female mate choice is, thus, possible in a variety of social organizations.

A second (and related) possible constraint on female choice is male behavior, which can limit a female’s access to sexual partners [2]. Male reproductive strategies may include mate guarding or female-directed aggression, both of which might prevent a female from safely seeking out other mates. A classic example of a species with male reproductive power is hamadryas baboons (*Papio hamadryas*), in which larger males use coercive tactics to control females in their one-male units [4]. Similarly, male Tonkean macaques guard fertile females and threaten them if they approach other males, potentially constraining female access to preferred mating partners [9]. Male red deer (*Cervus elaphus*) and North American elk (*Cervus canadensis*) aggressively exclude other males from their harems, limiting the males that females can safely access [5]. Chimpanzee males achieve higher copulation success with the females towards whom they are most aggressive, demonstrating strong male influence over mating outcomes [17]. In such situations of conflicting sexual interests between males and females, females may not be able to exercise choice freely or successfully. 

Male behavior is most likely to constrain female choice in groups with low reproductive synchrony. According to the priority of access model, high-ranking males can better monopolize reproduction when fewer females are fertile at once [18]. By contrast, high reproductive synchrony can prevent a single high-ranking male from successfully monopolizing all sexually receptive females, enabling females to exercise mate choice more freely. Various studies have confirmed that alpha male mating and/or siring success tends to decline as the number of fertile females in a group increases (e.g., Japanese macaques (*Macaca fuscata fuscata*) [19]; mandrills (*Mandrillus sphinx*) [20]; chimpanzees [21]; Barbary macaques (*Macaca sylvanus*) [22]; review [23]). In fact, actual male copulation and paternity rates are often even lower than the priority of access model would predict [22,24,25,26]. Female agency and choice, particularly in these situations of low monopolization potential, may explain such deviations.

Thirdly, female characteristics (such as age, parity, and dominance position relative to other females) may limit an individual female’s ability to ensure that her preferred mate is the sire of her offspring. Age and parity might affect a female’s fecundity (e.g., very young and very old females tend to have longer interbirth intervals than middle-aged females [27]), and, therefore, may influence how attractive she is to her preferred males [28]. High rank is often associated with better access to food resources, and higher-ranking females may likewise have better access to preferred partners [29]. 

Females who do have the opportunity to exercise unconstrained mate choice should be most selective during the periovulatory period (POP), when fertilization is most likely [30]. In several primates, females do change their mating strategies around ovulation. For example, Japanese macaques copulate with older, long-tenured males when conception is unlikely, but avoid mating with these less preferred males around ovulation. When conception is likely, females preferentially mate with males who have had a shorter tenure in the group, possibly to increase genetic diversity of offspring [31]. Similarly, female orangutans (*Pongo pygmaeus*) in the POP are more likely to resist non-prime, unflanged males, despite potentially high physical costs of resistance [32]. In chimpanzees, both lower rates of female proceptivity and higher rates of female resistance during the POP suggest that females are less promiscuous and more discriminating when conception is likely [30].

In this study, we investigated female mate choice in blue monkeys (*Cercopithecus mitis stuhlmanni*). Blue monkeys generally live in one-male groups, which can experience seasonal male influxes that last from several days to several months [33]. Resident males are more stable group members than males who join groups solely during influxes, but are still typically social outsiders (females interact with them primarily during the breeding season, and little at other times) [34]. Groups are female-philopatric, and adult females form stable matrilineal dominance hierarchies. Adult males dominate all females [35]. Though copulations can happen at any time, conceptions are seasonal, with most occurring between June and August in our study population; many females in a group are, thus, fertile and sexually active at the same time [36]. Blue monkey females do not have sexual swellings to advertise their fertility, and are known to copulate at times when they are unlikely to conceive [37].

Despite living in one-male groups, blue monkey females have opportunities to access multiple potential mates. As noted above, extra-group males may temporarily join groups during the breeding season, especially larger groups with many sexually active females, and resident males seem unable to prevent such influxes [33]. Females may also come into contact with resident males of neighboring groups during intergroup encounters [38]. Paternity data show that resident male siring success decreases when there are multiple sexually receptive females in a group at once, and when there are many competitor males present, suggesting that residents are unable to monopolize all available mates in these situations [16]. Non-resident blue monkey males can indeed be quite successful in siring offspring: one study [16] found that 39% of 111 blue monkey infants born over the course of 10 years were sired by non-resident males. These findings demonstrate that blue monkey females not only have opportunities to interact with males other than their resident, but that females do copulate and sire offspring with these males, providing a potential pathway to female reproductive control even within a male-biased power structure.

Some studies of female mate choice have drawn criticism for insufficiently considering the influence of male behavior on female mating decisions [39]. Indeed, in many species, coercive male tactics such as mate guarding and outright aggression are both common and costly to females [40], both in terms of energetic stress [17] and risk of injury [2]. In blue monkeys, however, male coercion seems uncommon—females can successfully ignore, avoid, and evade unwanted approaches by males [36]. Further, the fact that resident males do not fully monopolize reproduction confirms that females have opportunities to mate with (and, thus, to prefer) non-resident males, even if non-residents are present only briefly. Additionally, females initiate the majority of sexual interactions in this species, towards both resident and non-resident males [36]. Although blue monkey males reject many of these solicitations, they rarely respond aggressively to females, further demonstrating low rates of female-directed aggression [28]. These characteristics of blue monkey mating strategies provided us with two ways to measure female preference: female resistance rates to male sexual solicitations, and female sexual solicitations themselves.

We used data gathered over a long-term study to determine (1) if blue monkey females express mate choice, either by selectively rejecting some males’ advances or by preferentially soliciting sexual interactions with certain males, (2) which males females prefer, and (3) if observed female mate preferences are successful in influencing paternity. Because we were interested in paternity, and because female behavior during conceptive estrus should be most informative regarding female preference, we focused our analyses on the sexual interactions during females’ conceptive periods for the infants included in the study. 

We hypothesized that females would often prefer influx males to resident males, particularly when the resident had already held a long tenure. Previous observations suggest that unfamiliar males are attractive to female blue monkeys [36]. These observations are consistent with the anti-infanticide strategy of paternity confusion, in which a female mates with multiple males to minimize the risk that a given male would kill her infant if he takes over the group when her infant is still young enough to be vulnerable [37]. Blue monkey males are indeed known to commit infanticide following resident male takeovers, in accordance with the sexual selection hypothesis [41].

We also expected that, if female mate choice was effective, most infants would be sired by the mother’s preferred male. If female behaviors can successfully influence paternity, this correlation should exist regardless of a male’s status as resident or non-resident, i.e., we expected that preferred non-residents would be more likely to be sires than non-preferred non-residents. To account for the potential differences between resident and non-resident males, we controlled for differential availability of males, and limited some analyses to residents or non-residents only.

Lastly, we predicted that infants sired by preferred males would be more likely to survive, as has been shown for mice [42]. In particular, we expected to see this result for infants who were vulnerable to infanticide by an incoming male. Blue monkey infants are at risk of infanticide if they are less than six months old during a male takeover, or born during a new male’s first few months as resident [41]. Male influxes can also threaten young infants (<6 months). Because infanticide is so costly to mothers, higher likelihood of infant survival would be a valuable benefit of successful female mate choice.

## 2. Materials and Methods

### 2.1. Study Population

All data came from a long-term field study. The study population inhabited the Kakamega Forest (0°19 N, 34°52 E, 1580 m), a Kenyan rainforest with a high density of blue monkeys (192 individuals per km^2^ [43]). The population has been monitored by trained observers on a near daily basis since 1979, providing us with detailed demographic data [44]. Researchers identified individual monkeys based on natural features and observed animals during daily group and focal animal follows.

Our dataset encompassed the sexual interactions of 54 females during the years 2002–2011. During this 10-year period, the study population included 3–4 groups under observation at any one time, because of one group fission in 2005. Female group size ranged from 8–29 adult females, and the number of males in the group ranged from 1–11 males, for a total of 178 male–female dyads that could have interacted during the female’s conceptive period (i.e., the male and female were observed in the same group on at least one day of the female’s conceptive window). Paternity data (see below) came from 77 infants born to the 54 females over the course of the study [16]. Five of the females gave birth twice during the study period, and four females gave birth three times, and, therefore, were included in the analyses multiple times (once for each conceptive estrus period preceding the birth of an infant).

### 2.2. Behavioral Data Collection

Behavioral data collection was part of long-term population monitoring, and included daily census data, focal follows of adult females, and ad libitum data on social and sexual interactions. Observers prioritized focal follows of adult females, who are more stable group members than males, but also recorded all observed sexual interactions at any time in as much detail as possible. Sexual interactions included copulations (mounting and thrusting, with or without ejaculation), as well as sexual solicitations by males (approaching and soliciting the female with head flagging or persistent following) and proceptive behavior by females (lip-puckering, presenting the hindquarters, or persistent following [37]). 

We focused on sexual interactions during the periods in which females conceived infants. We estimated female conceptive periods using the 95% confidence interval for gestation length (176 ± 14 days [45]), counting back from the birthdate of her infant. We chose not to examine sexual interactions outside of this period, which would not have associated paternity data. 

One possible way to measure female preference is through female responses to male-initiated sexual interactions (both copulations and cases of male solicitation, as described above). To examine this measure, we looked at each sexual interaction initiated by a male towards a particular female, and noted whether the female accepted or resisted the behavior. Acceptances ultimately led to copulations, whereas resistance behaviors included screaming, avoiding, ignoring, and leaving. By this measure of female preference, a lower rate of female resistance towards a particular male would suggest that the female prefers him, whereas a higher rate of resistance would indicate that she does not prefer him. For this analysis, we focused exclusively on interactions between females and resident males to avoid large discrepancies in the number of male-initiated sexual interactions, which are likely a function of male power and/or presence (resident males are dominant to intruders, and also present in groups for more days, so may solicit females more often). Additionally, we excluded females from this analysis if no male-initiated sexual behavior was observed for them.

The second measure for female preference that we examined was unsolicited proceptive behavior. For each female, we specified this value for each male who was present in her group during any part of her 29-day conceptive window. To calculate this proportion for each male, we tallied the unsolicited proceptive interactions, or the instances in which the female directed proceptive behavior (as defined above) towards him, without his having initiated the interaction by approaching or soliciting her. We included all female-initiated proceptive behavior in this count, regardless of whether the behavior ultimately led to copulation. We considered subsequent behaviors between the same female and male, including additional proceptive signaling, to be part of a single interaction until either the female or male (or both) left each other (separated > 1 m) or stopped interacting with one another (if the proceptive interaction had occurred over a distance). We then divided the number of unsolicited proceptive interactions that the female had initiated towards a particular male (X) by the *total* number of proceptive interactions that she had directed towards *any* male in the group on the days when he (X) was present. Using this ratio corrected for the differential availability of males, which was necessary because residents were in groups more than non-resident males. We refer to this value as the “proportion of unsolicited proceptive behavior”:

(1) $$\begin{array}{c} \mathrm{proportion}\mathrm{of}\mathrm{unsolicited}\mathrm{proceptive}\mathrm{behavior}\\ =\frac{\#\mathrm{unsolicited}\mathrm{proceptive}\mathrm{interactions}\mathrm{the}\mathrm{female}\mathrm{directs}\mathrm{to}\mathrm{this}\mathrm{male}}{\#\mathrm{unsolicited}\mathrm{proceptive}\mathrm{interactions}\mathrm{the}\mathrm{female}\mathrm{directs}\mathrm{to}\mathrm{any}\mathrm{male}\mathrm{while}\mathrm{this}\mathrm{male}\mathrm{is}\mathrm{in}\mathrm{her}\mathrm{group}} \end{array}$$

After calculating this proportion for each female–male dyad, we were able to compare the values directly to identify the male or males that each female had solicited most frequently. If two or more males received the same score for a particular female and conceptive period, we considered neither male to be the single most solicited. 

From long-term life history and social data, we extracted characteristics that might influence a female’s ability to obtain her preferred mate as a sire, namely, her parity and dominance rank at the time of conception. We considered a female to be parous if she had given birth previously (regardless of infant survival), meaning that the infant she conceived and birthed in this study was not her first pregnancy. We assessed female dominance rank annually using the I&SI ranking method in DomiCalc [46], based on dyadic agonistic interactions between adult females with clear winners and losers. We expressed rank for each female on a scale of 0–1, with a rank of 1 meaning that she dominated all other adult females in her group, and a rank of 0 meaning that she was subordinate to all of her female peers [35]. Because rank was recalculated each year, and our study spanned multiple years, females present in our dataset more than once did not necessarily retain the same rank for each year they were included in the study. 

We also pulled information about male status from long-term records. We defined a resident male as one who had been the sole male in the group for at least 7 consecutive observation days. A male lost his status as resident when he left the group for at least 7 consecutive observation days. Any male who was not the current resident of the group he was in was considered a “non-resident” male for that day, even if he was a former resident or later became the resident of the group. We made one exception to this criterion for a male who was (1) the most consistently present male over an unusual four-year period when multiple males were present nearly every day, and (2) the male who produced nearly all “pyow” calls, which are not given by non-resident males [47]. (Without our making this exception, this male’s group would have no male that met the “resident” criteria during the four-year period).

For resident males, we also calculated the number of days that the male had been a regular part of the group, with no absences of more than two consecutive observation days. We chose this measure, rather than the number of days since the resident male’s first “official” day as resident, to more accurately represent females’ familiarity with and prior access to that male, even if he was not yet resident. 

To study infant survival, we used infant birth and death dates from the long-term study. We classified infants as vulnerable to infanticide if they were born six months before or after a resident male takeover. We also noted whether infants had experienced an influx situation (a period of at least seven consecutive days with multiple and/or novel males in the group) during their first six months of life, as the presence of so many non-resident males could also increase the infant’s risk. 

### 2.3. Paternity Assessment

We compared the identities of preferred males with paternity data to determine if the most solicited males were also assigned as fathers. Paternity data came from [16], who extracted and amplified microsatellite loci from fecal samples collected from infants, their mothers, and adult males. DNA extraction was performed using the QIAamp^®^ DNA Stool Mini Kit (Qiagen, Germantown, MD, USA), and DNA was amplified using the Qiagen^®^ Multiplex PCR Kit. The ABI 3730 Automated DNA Analysis system and GeneMapper 3.7 were used for genotyping, and likelihood-based paternity analysis was conducted with the software package Cervus 3.0 (see [16] for a full description of genetic analysis and paternity assignment). Males identified as fathers were assigned with 80–95% confidence.

### 2.4. Data Analysis

Data analysis was conducted in R [48]. We used mixed effects logistic regression (binomial error structure and logit link function, glmer() function, lme4 package [49]) to analyze the effect of female preference and male residency on paternity (i.e., whether the male sired the infant), first for all male–female pairs (N = 178), and then for non-resident male–female pairs only (N = 112). We also modeled the effect of female rank and parity on her siring offspring with her preferred male. Models included predictor variables (proportion of unsolicited proceptive behavior and male status as resident or non-resident, and female rank and female parity at the time of conception) as fixed effects, and male and female identities as random effects. We used likelihood ratio tests (LRTs) to evaluate predictors, comparing full models to null models with random effects only, or to reduced models that omitted one predictor variable that we were evaluating. Originally, we included interaction effects between predictor variables, but these were not significant and we dropped them to facilitate interpretation of main effects [50]. We assessed collinearity in predictors using variance inflation factors (VIFs), and used the DHARMa package for model diagnostics [51]. 

We used Fisher exact tests for analyses that examined associations between two nominal variables: the relationship between a male being resident of a female’s group and being most solicited by that female, between a female being parous and the female preferring a resident male, between a male being most solicited by a female and him siring that female’s infant, and between an infant being sired by its mother’s most preferred male and the infant surviving at least one year. We also used Mann–Whitney U tests to examine the relationship between the number of days a female’s resident male had been present in the group and her preference for resident or non-resident males, and between a female’s rank and her preference for resident or non-resident males.

## 3. Results

### 3.1. Female Resistance Rates

The first measure of female mate choice we investigated was female resistance to male-initiated sexual interactions. Male-initiated sexual behavior was observed in 23 resident male–female pairs. In all but two cases, females did not resist male advances, regardless of whether the male was the father. Male solicitations appear to be rarer than female-initiated sexual interactions in blue monkeys, so female resistance may simply be a less common way for females to express preference in this species. As such, we did not use this measure to compare to paternity results in further analyses.

### 3.2. Proportion of Unsolicited Proceptive Behavior

**Figure 1:**
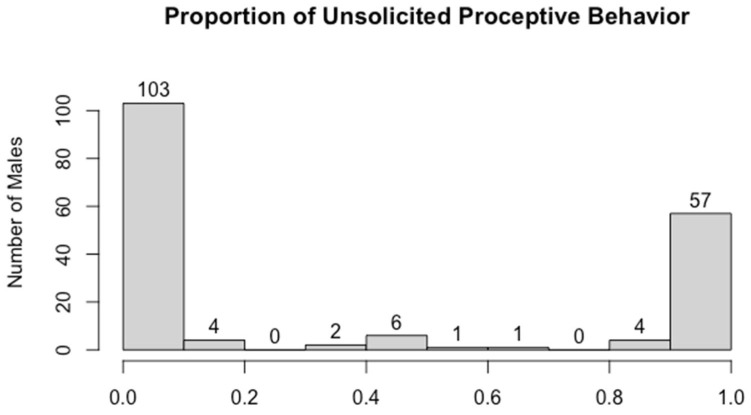
Histogram of the proportions of unsolicited proceptive behavior assigned to individual males (N = 178 male–female dyads).

Our second measure of female preference, the proportion of unsolicited proceptive behavior, ranged from 0–1 (median = 0, N = 178 male–female dyads, Figure 1). Most males earned a score of either 0 (57%) or 1 (32%), meaning that while the given male was in the group, the female either directed no proceptive behavior towards him, or all proceptive behavior towards him. In some cases, a score of 1 reflected a single unsolicited proceptive interaction directed toward that male, but more often, a score of 1 resulted from multiple proceptive interactions all directed toward the same male (and none directed towards any other males).

### 3.3. Female Preference for Resident Males

Based on the proportion of unsolicited proceptive behavior, we identified a female’s most solicited male for 60 of 77 infants with known paternity. In the instances when we could not identify the single most solicited male, it was either because the mother had solicited two males at equal rates (4 cases), or because the mother was not observed to be proceptive towards any male during her conceptive window (13 cases). 

The resident male was most solicited by 53 of the 60 females (88%). In 49 of these cases (82% of the total, or 92% of cases in which the resident male was most solicited), the female directed proceptive interactions exclusively towards the resident when he was in the group (i.e., the proportion of unsolicited proceptive behavior for the resident equaled 1). There was a strong association between a male being resident and being the most highly solicited partner (Table 1, Fisher exact test, *p* < 0.0001). Even among females who sired offspring with non-resident males (N = 19), 63% had solicited their residents at higher rates.

Seven females (12%) directed most proceptive behavior towards a non-resident male. In five of these cases, the most solicited male was one of many males who had been in and out of the group during an influx period. In the other two cases, the most solicited male was the resident of a neighboring group.

We wondered whether females might be more likely to prefer a non-resident based on their parity, rank, or the length of time the resident had been present in the group (indicating the female’s familiarity with and previous access to him). Neither female parity nor rank appeared to influence whether the female preferred a non-resident (parity: Table 2, Fisher exact Test, *p* = 0.36; rank: Table 3, Mann–Whitney U test, W = 161, *p* = 0.12). There was a statistically significant difference in the number of days a resident male had been present in the group for females who preferred a resident vs. females who preferred a non-resident (Table 4, Mann–Whitney U test, W = 359, *p* = 0.02); females who preferred non-residents tended to have resident males with longer tenures. We note, however, that the sample size for females who preferred non-residents is small.

One of the females (Sauc) who preferred a neighboring resident appeared in our dataset twice, so it was possible to compare her preferences during different conceptive periods. During the 2003 conceptive period, she preferred the resident male (Putn). Her next conceptive period occurred in 2005, shortly after her group fissioned and during a period in which there were many males in the group each day. During this later conceptive period, she preferred and sired offspring with the resident male of a neighboring group (PH), and not the resident male of her own group (Rock). The other six females appeared in our dataset only once, so we were not able to evaluate whether their preferences for non-residents were consistent over time.

### 3.4. Predictors of Paternity

We were able to compare the proportion of unsolicited proceptive behavior with paternity for 55 infant births. We had already dropped 13 cases in which the mother was not observed to exhibit proceptive behavior during her conceptive period. Nine more cases were excluded because the assigned father was not detected in the female’s group during her conceptive window, so it was not possible to calculate the proportion of unsolicited proceptive behavior for him.

As expected, males who received the highest proportion of unsolicited proceptive behavior from a female tended to sire that female’s infant: the most solicited male was the father in 82% of cases (N = 55). Overall, males who received higher proportions of unsolicited proceptive behavior from particular females tended to be the fathers of those females’ infants, and those who received lower proportions of such behavior tended not to be fathers (N = 178 male–female dyads, Table 5, Fisher exact test *p* < 0.0001). The GLMM (Table 6), which included no interaction term, differed significantly from a null model with only random effects (LRT, *χ*^2^ = 24.5, 2 df, *p* < 0.0001). The proportion of unsolicited proceptive behavior had a clear effect on the male being the infant’s father (LRT, *χ*^2^ = 21.5, 1 df, *p* < 0.0001). The odds of siring (vs. not siring) a female’s infant increased over 26 times for males who had received all of a female’s unsolicited proceptive behavior vs. those that had received none of her unsolicited proceptive behavior (Table 6). By contrast, male status (resident vs. not) did not have a clear effect on paternity. VIFs were <1.06, showing that the predictors were not collinear. Diagnostic tests performed using DHARMa further indicated that the model was a good fit (Figure S1, Table S1). 

To confirm the independent effect of female preference on likelihood of paternity, we reran the model on a dataset of non-resident males only (N = 112 male–female dyads involving non-resident males). This GLMM showed that among non-residents, a male’s proportion of unsolicited proceptive behavior was still a strong predictor of him fathering the infant (odds ratio: 33.59, SE: 0.94, 95% CI: 31.75–35.43). The odds that a most solicited non-resident male sired (vs. did not sire) that female’s infant were over 30 times greater than those of a non-preferred non-resident male siring the offspring. A likelihood ratio test confirmed that this GLMM differed significantly from a null model (χ^2^ = 14.2, 1 df, *p* < 0.0002), and uniformity and dispersion tests also suggested goodness of fit (Figure S2, Table S1).

### 3.5. Paternity by Non-Preferred Males

Females successfully sired offspring with their most solicited male in most cases; however, in 15 of 60 cases (25%), the infant’s father was not the male who received the highest proportion of unsolicited proceptive behavior from the infant’s mother. Neither female rank nor parity at the time of conception was a significant predictor of whether the infant’s father was the female’s most solicited partner (Table 7), and the GLMM did not differ significantly from a null model (*χ*^2^ = 0.45, 2 df, *p* = 0.80).

### 3.6. Infant Survival

An infant’s survival to one year was not clearly related to its father having been most preferred by its mother. In total, 5 of the 55 infants in the study (9%) died before their first birthday. Four were sired by their mother’s most preferred male, and one was sired by a less preferred male (Table 8). Preferred sires did not appear to produce more resilient infants (Fisher exact test, two-tailed *p* = 1), but the sample of deaths was small.

Seven of the infants in our study (13%) were vulnerable to infanticide because a resident male takeover had occurred in their group within six months of their birth. Of these infants, three were born in the six months preceding a takeover, and the other four were born up to six months after a takeover. An additional 16 infants (29%) were vulnerable to infanticide because of an influx situation, with multiple novel males in their group when they were less than six months old. In total, 23 infants (42%) were at risk of infanticide based on at least one of these factors. 

When we analyzed this subset of 23 vulnerable infants, we once again found no significant association between preferred sires and the survival of offspring: 2 of the 15 infants sired by preferred males died, and 0 of the 8 infants sired by nonpreferred males died (Fisher exact test, two-tailed *p* = 0.53). Again, however, the sample size was small.

## 4. Discussion

### 4.1. The Efficacy of Female Mate Choice

Female mate choice in blue monkeys, as measured by the proportion of unsolicited proceptive behavior that females directed towards each male, seems to be effective: 82% of infants were sired by the mother’s most frequently solicited male, without apparent male coercion or aggression, and female preference for a particular male was a significant predictor of his siring the infant. Males who were most frequently solicited tended to be fathers, even if they were not residents, suggesting that female blue monkeys are not as limited by male dominance as many other primate females. Overall, our results provide potential evidence that females can successfully influence reproductive outcomes, even in one-male polygynous groups. In other words, we argue that female blue monkeys may have some degree of intersexual power (namely, control over the identities of sires), despite the male-biased power structure of blue monkey groups. It seems likely that this power derives from breeding seasonality combined with the visually dense environment in which these monkeys live, making it possible for a female to mate with her chosen partner while evading the vigilance of her resident male if necessary. Resident males were more often fathers, but not *because* they were residents (male status was not a significant predictor of paternity). It appears instead that these males are more likely to be fathers because these are the males that females prefer.

### 4.2. Female Preference for Resident Males

Contrary to our expectation, 88% of females directed most unsolicited proceptive behavior to their resident male. Blue monkey resident males do not provide paternal care, nor do they generally protect individual females, so we had expected females to prefer to copulate with extra-group males as a part of a strategy to avoid infanticide. While infanticidal behavior following resident male takeovers does occur in blue monkeys, it is highly variable, depending largely on individual male identity [41]. Furthermore, the position of resident male is itself dependent upon successful male–male competition; a male’s status as resident indicates his ability to outcompete other males, possibly because of his superior qualities [5,38,52]. Perhaps the fact that resident males must have already out-competed rivals makes them more attractive to females [52], or perhaps females benefit more from focusing their attention on stable resident males, who have already demonstrated competitive ability, rather than non-residents who may not succeed in taking over the group. 

Because females largely preferred resident males (i.e., solicited them at higher rates), it is not possible to fully separate our results from the impact of male–male competition. However, if resident males are indeed of higher quality, then female choice for residents is equivalent to female choice for superior mates, and resident males’ siring success is an indicator of effective female choice. Our additional analysis of non-resident males, which showed that more frequently solicited non-residents were more likely to be sires than nonpreferred non-residents, further supports the conclusion that female mate choice is effective regardless of male status. 

Nevertheless, the tendency to prefer resident males may not be uniform across females or consistent over time. Most females did solicit resident males at higher rates than non-residents, but 7 of 60 females (12%) seemed to prefer an extra-group male. Although nulliparous females are perhaps less attractive to resident males [28], and low-ranking females might compete less successfully to access resident males [29], we did not find an association between either factor and female preference for non-residents. There was, however, a negative association between a female’s preference for her resident and the length of time that resident had been present in her group, suggesting that females prefer novel males over residents with whom they have had extensive access and multiple opportunities to mate. This finding is consistent with previous observations of this study population [36], and with other studies on mammals that have reported female preference for unfamiliar males or males who are earlier in their tenures [10,53,54].

Notably, most of the females who preferred non-residents did so during a time in which multiple males were present in the group, perhaps increasing their exposure to males other than their resident, and providing them with opportunities to both assess and interact with these potential mates. The female who appeared in our dataset twice, once preferring her resident and once preferring a non-resident, preferred the non-resident shortly after a group fission. These observations, though few, are consistent with the hypothesis that females mate promiscuously during periods of potential instability [55,56]. 

Overall, these results suggest that social context is an important factor in influencing female mate preferences. Female blue monkeys seem to prefer residents as a default, but they prefer non-residents in certain social contexts (if they have already had a lot of exposure to and opportunities to mate with their resident, or when they might benefit from promiscuity).

### 4.3. Paternity by Non-Preferred Males

For the small number of cases in which a female’s most solicited male was not the father of her infant, we found that neither her parity nor rank predicted her success at obtaining her preferred sire. Though parity can provide information about female fertility, there is mixed evidence regarding its influence on reproductive success overall. Parous females may be more attractive to males in some situations [55,57], but parity is not a consistent predictor of a male responding positively to a female’s sexual solicitation [28]. Parity is also not a meaningful predictor of blue monkey infant survival [58]. 

The fact that a female’s rank did not affect her success in obtaining her preferred sire is consistent with other studies investigating the effects of dominance on blue monkey female behavior and reproduction. High-ranking females are by definition more successful in agonistic intrasexual interactions, which could provide them with greater access to items over which they compete directly. Blue monkey females compete directly over food [59], but there is mixed evidence on how dominance rank impacts the diet: one study reported that high-ranking females tend to feed on preferred, energy-rich foods such as fruits more than lower-ranking individuals [60], although another found that higher-ranking females do not meet nutritional intake targets more closely [61]. Dominance rank also does not appear to influence reproduction in blue monkey females: it does not accurately predict probability of conception [44], nor does high rank increase the likelihood that a male will accept a female’s sexual solicitation [28]. Similarly, as we report here, rank does not predict whether a female will be successful at executing mate choice. 

### 4.4. Infant Survival

We found no evidence that female preference for an infant’s father was associated with the infant’s survival, even when we controlled for the infant’s vulnerability to infanticide. This may be because infanticide is not the only cause of infant death. An earlier study on this population found that 28% of infants died before their first birthday, with known or suspected causes of deaths including infanticide by males, direct or indirect interactions with humans (such as coming into contact with snares), and an interaction with a hawk [58]. Many other individuals simply disappear and their exact cause of death is unknown. Because survivorship is influenced by so many factors, it is unsurprising that sire identity does not play a larger role in determining whether an infant lives or dies.

## 5. Conclusions

Despite a social organization that would seem to favor male control of reproduction, female blue monkeys seem to be active participants in their sexual interactions. They solicit copulations from desired partners and are largely successful in siring offspring with preferred males, even when those males are not the resident male in their modally one-male groups. Social context appears to be a factor in female mating preferences. Though we could not entirely discount the confounding effects of male–male competition on observed female behavior and paternity, we found that female preference predicted paternity even among non-resident males (who have not succeeded in intrasexual competition for group residency). Overall, males were significantly more likely to be sires if females preferred them, regardless of status. As such, blue monkeys provide a potential example of successful female mate choice, even in a species with male-biased intersexual power.

## Figures and Tables

**Table 1:** Distribution of resident and most solicited males.

# of Cases	Resident	Non-Resident
Male is most solicited	53	7
Male is not most solicited	11	107

**Table 2:** Female parity and preference for non-resident males.

# of Cases	Parous Female	Nulliparous Female
Female prefers resident	44	9
Female prefers non-resident	4	3

**Table 3:** Female rank and preference for non-resident males.

	Median Rank	IQR	Sample Size
Female prefers resident	0.57	0.48–0.83	53
Female prefers non-resident	0.33	0.14–0.58	7

**Table 4:** Resident male tenure in the group and female preference for non-resident males.

	Median Resident Male Tenure (Days)	IQR	Sample Size
Female prefers resident	415	37–912	53
Female prefers non-resident	1140	769–1357	7

**Table 5:** Distribution of males who were most solicited and fathers.

# of Cases	Male Is Father	Male Is Not Father
Male is most solicited	45	15
Male is not most solicited	10	108

**Table 6:** Generalized linear mixed model predicting whether a given male sired a given infant as a function of the proportion of unsolicited female proceptive behavior he received from the infant’s mother and his status as resident (N = 178 male–female dyads). Random effects were male ID and the ID of the infant’s mother ^1^. LRT compares full model to model minus each predictor.

	Odds Ratio	SE	95% CI	LRT
Proportion of unsolicited proceptive behavior	26.64	0.73	25.21–28.07	χ^2^ _df=1_ = 21.5, *p* < 0.0001
Male resident ^2^	3.92	1.85	0.29–7.55	χ^2^ _df=1_ = 0.55, *p* = 0.46

^1^ Variance of random effects: male ID 2.58, female ID < 0.001. ^2^ Reference class: not resident.

**Table 7:** Generalized linear mixed model predicting if a female sired offspring with her most solicited male as a function of her rank and parity at the time of conception (N = 60 mothers). Random effects were male and female ID*. LRT compares full model to model minus each predictor.

	Odds Ratio	SE	95% CI	LRT
Rank	2.38	1.40	−0.36–5.12	χ^2^ _df=1_ = 0.393, *p* = 0.53
Parity	1.03	0.93	−0.80–2.86	χ^2^ _df=1_ = 0.001, *p* = 0.97

* Variance of random effects: male ID 1.40, female ID < 0.001.

**Table 8:** Distribution of infants who survived past one year of age.

# of Cases	Infant Survived	Infant Did Not Survive
Infant sired by preferred male	41	4
Infant sired by non-preferred male	9	1

## Data Availability

The data used in the study will be openly available on Dryad at https://doi.org/10.5061/dryad.d2547d896.

## Supplementary Materials

The following supporting information can be downloaded at: https://www.mdpi.com/article/10.3390/ani14111589/s1, Figure S1: QQ and residual plots produced with DHARMa for Model 1 (N = 178); Figure S2. QQ and residual plots produced with DHARMa for Model 2 (N = 112); Table S1: DHARMa nonparametric dispersion test for each model.
