# Supplementary material for: Female Mate Choice in Wild Kenyan Blue Monkeys (Cercopithecus mitis)
Source: Animals (Basel). 2024 May 28;14(11):1589. doi: 10.3390/ani14111589 (PMC11171077; doi:10.3390/ani14111589)
Supplement: Supplementary file 1 [file animals-14-01589-s001.zip › animals-2997229-supplementary.pdf]

Supplemental Information

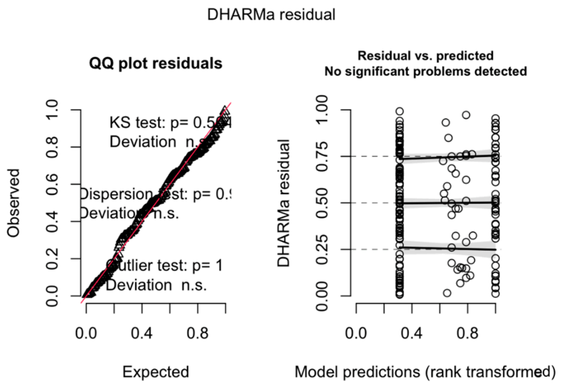

Figure S1. QQ and residual plots produced with DHARMa for Model 1 (N=178).

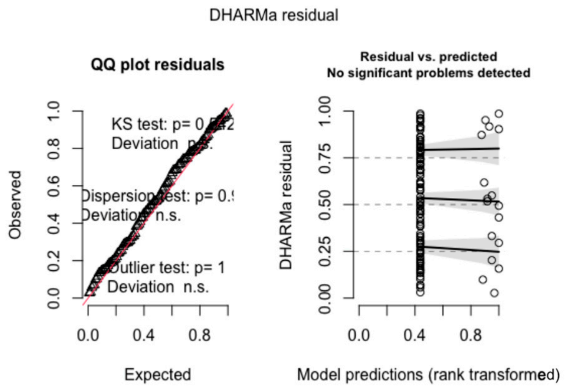

Figure S2. QQ and residual plots produced with DHARMa for Model 2 (N=112).

Table S1. DHARMa nonparametric dispersion test for each model.

Model	Dispersion	P-value
Model 1	0.84	0.96
Model 2	1.01	0.92
